# Disciplining a Rubidium Atomic Clock Based on Adaptive Kalman Filter

**DOI:** 10.3390/s24144495

**Published:** 2024-07-11

**Authors:** Kun Liu, Xiaolong Guan, Xiaoqian Ren, Jianfeng Wu

**Affiliations:** 1 National Time Service Center, Chinese Academy of Sciences, University of Chinese Academy of Sciences, Xi’an 710600, China; liukun@ntsc.ac.cn (K.L.); guanxl@ntsc.ac.cn (X.G.); renxq@ntsc.ac.cn (X.R.); 2 University of Chinese Academy of Sciences, Beijing 100049, China; 3 Key Laboratory of Time Reference and Applications, Chinese Academy of Sciences, Xi’an 710600, China

**Keywords:** adaptive Kalman filter, rubidium atomic clock, autocovariance least squares, time and frequency, Allan deviation

## Abstract

Rubidium atomic clocks have been used extensively in various fields, with applications such as a core component of Global Navigation Satellite Systems (GNSS). However, they exhibit inherently poor long-term stability. This paper presents the development of a control system for rubidium atomic clocks. It introduces an adaptive Kalman filtering algorithm for the disciplining of a rubidium atomic clock, utilizing autocovariance least squares (ALS) to estimate the clock’s noise parameters. The experimental results demonstrate that the proposed algorithm achieves a high estimation accuracy. The standard deviation of the clock error between the steered rubidium atomic clock 1 Pulse Per Second (1PPS) and Coordinated Universal Time (UTC) provided by the National Time Service Center (NTSC) is better than 2.568 nanoseconds(ns), with peak-to-peak values improving to within 11.358 ns. Notably, its frequency stability is reduced to 3.06 × 10^−13^ @100,000 s. The results for the rubidium atomic clock demonstrate that the adaptive Kalman filtering algorithm proposed herein constitutes an accurate and effective control strategy for the rubidium atomic clock discipline.

## 1. Introduction

Atomic clocks play an indispensable role in various critical domains, such as high-precision navigation and positioning [1,2,3,4,5], power grids [6,7], time synchronization [8,9], and communication [10,11]. Currently, the prevalent types of atomic clocks include hydrogen atomic clocks, cesium atomic clocks, and rubidium atomic clocks, each with distinct features and advantages suited to different application scenarios. Hydrogen atomic clocks and cesium atomic clocks excel in terms of frequency stability and daily drift rate, rendering them ideal for applications demanding the utmost precision and stability, such as maintaining national standard time [12]. However, these two atomic clocks are expensive and require stringent environmental conditions [13]. Conversely, rubidium atomic clocks boast the advantages of lower cost, compact size, high portability, and minimal power consumption [14]. In fields related to time synchronization in communication, navigation, and power grid systems, rubidium atomic clocks are broadly adopted due to their well-rounded performance characteristics.

Rubidium atomic clocks often suffer from poor long-term stability due to frequency drift and aging. Thus, in order to enhance the frequency stability of the rubidium atomic clock, researchers have adopted two primary approaches. The first approach involves internal adjustments to the rubidium atomic clock to improve the frequency drift. For example, Yueyang Wu et al. used a 780 nm external cavity diode laser (ECDL) as a seed source, locking the frequency to the rubidium atomic absorption peak to achieve high-frequency stability. A new frequency-locking algorithm was employed to eliminate frequency jumps caused by light shift effects, enhancing both precision and long-term stability [15]. Ganghua Mei and his team at the Chinese Academy of Sciences developed a new rubidium atomic clock using xenon as the starter gas in a rubidium spectral lamp with optical and isotopic dual filtering techniques, achieving 100 s frequency stability of 9 × 10^−15^ [16]. The second research approach involves locking the rubidium atomic clock 1PPS signal to a more stable reference 1PPS signal, such as the GPS 1PPS. This study is based on the second approach, using external reference 1PPS to enhance the long-term stability of the rubidium atomic clock.

Compared to that of the rubidium atomic clock, the long-term stability of the 1PPS signal output by GPS receivers is superior [17]. Locking to the reference 1PPS requires estimating the frequency deviation and aging through clock difference data. Xiaohui Li and colleagues used the least squares (LS) method to estimate the parameters of the rubidium atomic clock and employed the ping-pong algorithm to adjust its frequency [18]. However, this approach requires substantial clock difference data and exhibits significant system latency. S. R. Stein introduced the use of Kalman filtering for real-time estimation of atomic clock parameters, where the noise and covariance are determined using noise spectral density values [19]. However, it is necessary to pre-determine the Allan variance in the clock difference. Steven Hutsell discussed the shortcomings of using the Allan variance for rubidium atomic clock parameter estimation and proposed using the Hadamard variance to estimate the noise parameters in the Kalman filter equations [20]. D. A. Howe et al. suggested that the total Hadamard variance can be used to estimate the frequency stability of rubidium atomic clocks [21], thereby enhancing the reliability of long-term estimates, although this increases the computational burden. Duosheng Fan and colleagues designed a Kalman filter control algorithm for rubidium atomic clocks based on total Hadamard variance. They performed disciplining and analysis of rubidium atomic clocks with related equipment [22]. However, determining the noise parameters of rubidium atomic clocks using the Hadamard variance or Hadamard total variance requires multiple differencing of clock difference data, which is computationally intensive. To address the limitations of current algorithms, this paper proposes an adaptive Kalman filtering algorithm for the rubidium atomic clock disciplining directly from the Kalman filter model of the rubidium atomic clock. The ALS method is used to estimate the noise parameters of the rubidium atomic clock. Based on the constructed rubidium atomic clock control system, efficient and precise control of the rubidium atomic clock is achieved, effectively validating the accuracy and reliability of the proposed adaptive Kalman filtering algorithm.

## 2. Basic Principle and Control System

### 2.1. Kalman Model for Rubidium Atomic Clocks

The discipline of a rubidium atomic clock is inseparable from a precise clock difference model. Typically, the state parameters to be estimated for a rubidium atomic clock include the clock difference, frequency deviation, and frequency drift. Consequently, quadratic polynomial models of clock differences are commonly employed in rubidium atomic clock discipline algorithms. The clock differences between the rubidium atomic clock 1PPS and the reference 1PPS from the GPS conform to the following quadratic polynomial relationship.

(1) $$T\left(t\right)=x+yt+\frac{1}{2}zt^{2}+\varepsilon_{x}(t)$$

where x denotes the initial clock difference corresponding to the reference 1PPS, y represents the frequency deviation between the rubidium atomic clock and the reference frequency, and z signifies the frequency drift. The clock difference, frequency deviation, and frequency drift are all deterministic components. The quantity T(t) denotes the clock difference at time t. The term $\varepsilon_{x}(t)$ signifies the state noise of the clock difference, constituting the stochastic component. Notably, only the clock difference can be directly measured among these elements.

Using Equation (1), it is not difficult to construct the state equation for the rubidium atomic clock.

(2) $$\left[\begin{array}{c} x(t+\tau )\\ y(t+\tau )\\ z(t+\tau ) \end{array}\right]=\left[\begin{array}{ccc} 1 & \tau & \tau^{2}/2\\ 0 & 1 & \tau \\ 0 & 0 & 1 \end{array}\right]\cdot \left[\begin{array}{c} x(t)\\ y(t)\\ z(t) \end{array}\right]+\left[\begin{array}{c} \Delta x\\ \Delta y\\ \Delta z \end{array}\right]$$

In the state equation, τ represents the sampling time interval. Meanwhile, x(t), y(t) and z(t) represent the clock difference, frequency deviation, and frequency drift at time t, respectively. The terms Δx, Δy, and Δz represent random errors in the model, each with a mean of zero and uncorrelated with the rubidium atomic clock’s state variables. Discretization is performed on Formula (3):

(3) $$X(k+1)=AX(k)+w_{k}$$

In the formula, X(k+1) represents the three-dimensional state vector at time $t_{k+1}$, while X(k) denotes the three-dimensional state vector at time $t_{k}$. The vector $w_{k}$ signifies the error vector in three dimensions, with its covariance matrix denoted as Q.

(4) $$Q=E[w_{k}w_{k}^{T}]=\left[\begin{array}{ccc} q_{1}\tau +q_{2}\tau^{2}+q_{3}\tau^{5}/20 & q_{1}\tau^{2}/2+q_{3}\tau^{4}/8 & q_{3}\tau^{3}/6\\ q_{2}\tau^{2}/2+q_{3}\tau^{4}/8 & q_{2}\tau +q_{3}\tau^{3}/3 & q_{3}\tau^{2}/2\\ q_{3}\tau^{3}/6 & q_{3}\tau^{2}/2 & q_{3}\tau \end{array}\right]$$

Here, $q_{1}$, $q_{2}$, and $q_{3}$ are the noise variances associated with the state error vector $w_{k}$, representing the variances in phase white noise, frequency white noise, and frequency random walk noise, respectively. The term τ, as previously mentioned, represents the time interval. A denotes the state transition matrix.

(5) $$A=\left[\begin{array}{ccc} 1 & \tau & \tau^{2}/2\\ 0 & 1 & \tau \\ 0 & 0 & 1 \end{array}\right]$$

With τ fixed, A becomes a constant coefficient matrix. The observation equation for the rubidium atomic clock is represented as follows:

(6) $$Z_{k}=HX(k)+v_{k}$$

Within the parameter model of the rubidium atomic clock, only the clock difference is observable; hence, $Z_{k}$ is a one-dimensional observation vector. The state observation matrix $H=\left[\begin{array}{ccc} 1 & 0 & 0 \end{array}\right]$ is a constant coefficient matrix. The term $v_{k}$ denotes the observation noise, characterized by a zero mean and a variance in R.

(7) $$R=E\left[v_{k}v_{k}^{T}\right]$$

For the sake of simplicity, it is typically assumed that the state noise $w_{k}$ and the observation noise $v_{k}$ are statistically independent. Formulas (2) and (6) establish the linear model for the rubidium atomic clock Kalman filter.

### 2.2. Methods for Adjusting Rubidium Atomic Clock

Rubidium atomic clocks are typically adjusted using two methods: frequency and phase adjustments. While phase adjustments can lead to abrupt changes in time, frequency adjustments offer a dual benefit. They maintain phase continuity and allow for the correction of both frequency offsets and phase discrepancies. To maintain uninterrupted phase output, this paper selects frequency adjustment as the preferred control method for the rubidium atomic clock.

The process of disciplining a rubidium atomic clock is a continuous one, designed to stabilize its phase and frequency. By continuously monitoring the clock’s performance, the control system fine-tunes the frequency and phase. This meticulous regulation enhances the clock’s precision and stability, ensuring the delivery of a reliable and consistent time-frequency signal.

### 2.3. Ping-Pong Algorithm

The ping-pong algorithm is a straightforward and effective frequency control method. It determines the frequency adjustment amount by utilizing the current clock offset value, the predicted clock offset value at the next moment, and the current frequency deviation, thereby maintaining the clock offset within a specified range. Within the context of the designed control system and the frequency control of the rubidium atomic clock, the ping-pong algorithm proves to be more resource-efficient compared to a PI controller. It demonstrates robust performance, effectively compensating for environmental changes and equipment aging that impact frequency stability. Conversely, a PI controller requires precise tuning of the proportional and integral gains to achieve optimal performance, is sensitive to noise, and demands more computational resources.

1. The measured clock offset and the predicted clock offset have the same sign, and the direction of the clock offset change approaches zero:

(8) $$\Delta f=\gamma \times t_{pre}/T$$

where Δf is the adjustment amount, $t_{pre}$ is the predicted value, γ=0.8 is the attenuation factor, and T is the time interval.

2. The current clock offset and the predicted clock offset at the next moment have the same sign, and the trend of the clock offset change moves away from zero:

(9) $$\Delta f=\gamma \times t_{current}/T+f_{current}$$

where $t_{current}$ and $f_{current}$ represent the clock offset and frequency value at the current moment, respectively.

3. The current clock offset and the predicted clock offset at the next moment have opposite signs. In this case, the ideal adjustment amount is to reduce the clock’s rate to zero:

(10) $$\Delta f=-\gamma \times f_{current}$$

### 2.4. Control System

**Figure 1:**
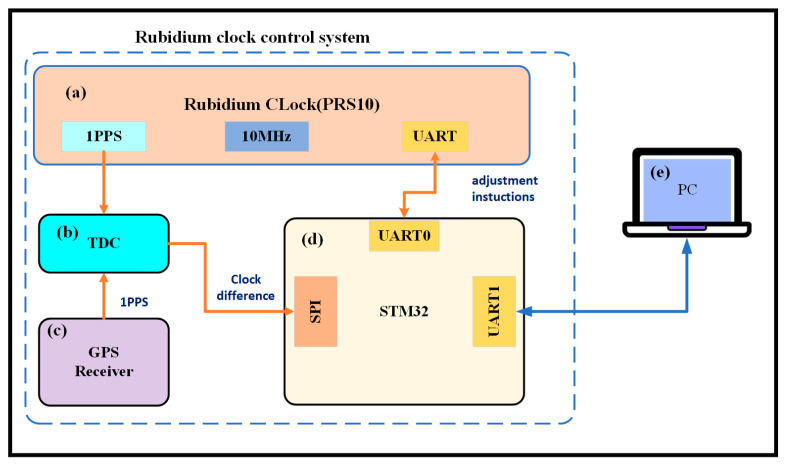
Diagram of the internal connections of the rubidium atomic clock control system (**a**) PRS10. (**b**) Counter based on TDC and FPGA. (**c**) GPS receiver. (**d**) STM32 F407. (**e**) PC.

**Figure 2:**
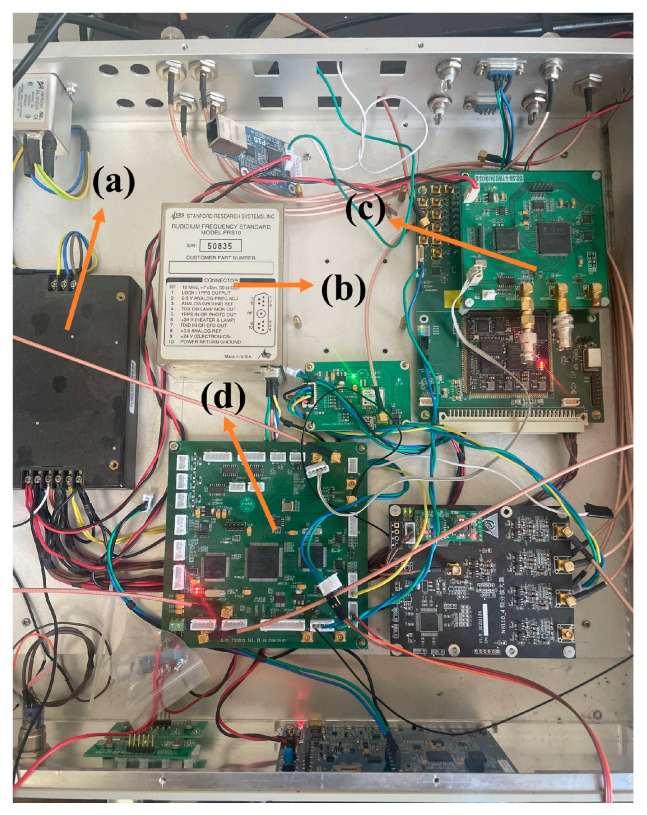
Diagram of the main components of the rubidium atomic clock control system. (**a**) Power supply. (**b**) PRS10. (**c**) Counter. (**d**) STM32 F407.

The internal connection diagram of the rubidium atomic clock control system is depicted in Figure 1. This system is mainly composed of four fundamental modules that handle data reception, processing, and the dispatch of control signals. The PRS10, a controllable rubidium atomic clock engineered by Stanford Research Systems (SRS) in the United States, is utilized within the system. A GPS receiver, crafted by the NTSC, captures the 1PPS signal emitted from GPS satellites. The measurement of the clock discrepancy is conducted by a time interval counter that leverages an FPGA-based platform and a time-to-digital converter (TDC) chip. This counter boasts a measurement precision of 25 picoseconds (ps), adequate for the calibration of the rubidium atomic clock. The microcontroller at the heart of the system is an STM32, equipped with a Cortex_M4 core. It refines the temporal discrepancy data via an AKF, thereby estimating the state parameters of the rubidium atomic clock. Guided by these estimations, the microcontroller transmits pertinent frequency adjustment commands to the rubidium atomic clock through a serial port, thereby modulating its oscillatory frequency. See Figure 2.

## 3. Description of the Algorithm

The adjustment of the rubidium atomic clock frequency is based on the estimation result of the state vector. Before accurately estimating the state variables of the rubidium atomic clock using the Kalman model, a key step is to determine the noise parameters in the model. Noise parameter estimation methods for the AKF primarily include Bayesian estimation, maximum likelihood methods, covariance matching methods, and correlation methods [23]. For linear time-varying models, the ALS method can estimate both state noise parameters and process noise parameters [24]. Compared to traditional algorithms, the ALS method significantly improves estimation performance and has achieved favorable results in the application of adaptive Kalman filters [25].

### 3.1. ALS Method

It is essential to determine the process noise covariance matrix Q and the measurement noise covariance matrix R. According to the mathematical model derivation, the value of the matrix Q is solely dependent on the time interval and the noise coefficient vector q, where $q=\left[\begin{array}{ccc} q_{1} & q_{2} & q_{3} \end{array}\right]$. In the noise density power model, $q_{1}$, $q_{2}$, and $q_{3}$ represent white phase noise, white frequency noise, and random walk frequency noise, respectively [26]. Appropriate noise parameters ensure that the filtering process accurately reflects the true fluctuations of the clock difference and effectively distinguishes signal changes from noise interference. If these parameters are not chosen appropriately, the noise covariance matrix will significantly impact the convergence performance. The Kalman model for the rubidium atomic clock, which is a linear filter model, reaches a steady state where the Kalman gain converges and can be represented by a constant vector K. Therefore, it is not difficult to construct the following innovation state-space model:

(11) $$\left\{\begin{array}{c} \varepsilon_{k+1|k}=A\varepsilon_{k|k-1}+Gw_{k}\\ Y_{k}=H\varepsilon_{k|k-1}+v_{k} \end{array}\right.$$

In this context, $\varepsilon_{k|k-1}$ denotes the prediction state error at time $t_{k}$, where $\bar{A}=A-AKH$, $\bar{G}=\left[\begin{array}{cc} G & -AK \end{array}\right]$ and $Y_{k}$ represent innovations in the Kalman filtering process. Notably, within the rubidium atomic clock Kalman filtering model, G is the third-order identity matrix.

The covariance matrix P is defined as $E\left[\varepsilon_{k|k-1}\varepsilon_{k|k-1}^{T}\right]$. P is obtained by the following Riccati equation.

(12) $$P=APA^{T}+GQG^{T}-AH^{T}{\left(HPH^{T}+R\right)}^{-1}HPA^{T}$$

When the Kalman filter reaches a steady state, the covariance matrix *P* also converges and satisfies the Lyapunov equation.

(13) $$P=\bar{A}P{\bar{A}}^{T}+R_{v}$$

Here, $R_{v}=\bar{G}\bar{Q}{\bar{G}}^{T}$, and $\bar{Q}=\left[\begin{array}{cc} Q & 0\\ 0 & R \end{array}\right]$. By employing vectorization operations, the matrix P can readily be transformed into its vector form:

(14) $$P_{s}={\left(I-\bar{A}⊗\bar{A}\right)}^{-1}\left[(G⊗G)Q_{s}+(AK⊗AK)R\right],$$

where $Q_{s}=Mq$. The expression for M is as follows:

(15) $$M={\left[\begin{array}{ccccccccc} \tau & 0 & 0 & 0 & 0 & 0 & 0 & 0 & 0\\ \tau^{3}/3 & \tau^{2}/2 & 0 & \tau^{2}/2 & \tau & 0 & 0 & 0 & 0\\ \tau^{5}/20 & \tau^{4}/8 & \tau^{3}/6 & \tau /8 & \tau^{3}/3 & \tau^{3}/2 & \tau^{3}/6 & \tau^{2}/2 & \tau \end{array}\right]}^{T}$$

Utilizing the above expression, Equation (15) can be restated as follows:

(16) $$P_{s}={\left(I-\bar{A}⊗\bar{A}\right)}^{-1}\left[(G⊗G)Mq+(AK⊗AK)R\right]$$

Equation (16) establishes a linear relationship between the error covariance vector $P_{s}$ and the state noise parameters $q_{1}$, $q_{2}$, and $q_{3}$, as well as the observation noise variance R. Odelson et al. defined the autocovariance matrix as follows:

(17) $$R(N)=E\left[\begin{array}{ccc} Y_{k}{Y_{k}}^{T} & \cdots & Y_{k}{Y_{k+N-1}}^{T}\\ \vdots & \ddots & \vdots \\ Y_{k+N-1}{Y_{k}}^{T} & \cdots & {Y_{k+N-1}}^{T}{Y_{k+N-1}}^{T} \end{array}\right]$$

Here, N denotes the lag order. The first column of the autocovariance matrix represents the autocorrelation sequence of innovation $Y_{K}$.

(18) $$R_{1}(N)=E\left[\begin{array}{c} Y_{k}{Y_{k}}^{T}\\ \vdots \\ Y_{k+N-1}{Y_{k}}^{T} \end{array}\right]$$

By applying Equation (18), it is straightforward to derive the subsequent equation.

(19) $$\begin{array}{l} E(Y_{k}Y_{k}^{T})=CPC^{T}+R_{v}\\ E\left(Y_{k+N}Y_{k}^{T}\right)=H{\bar{A}}^{N}PH^{T}-H{\bar{A}}^{N-1}AKR_{v}\;N\geq 1 \end{array}$$

By combining Equations (13), (18) and (19), the representation of $R_{1}(N)$ can be conveniently derived as follows:

(20) $$R_{1}(N)=\underset{O}{\underset{︸}{\left[\begin{array}{c} H\\ H\bar{A}\\ \vdots \\ H{\bar{A}}^{N-1} \end{array}\right]}}PC^{T}+\underset{\Gamma }{\underset{︸}{\left[\begin{array}{c} I\\ -HAK\\ \vdots \\ -H{\bar{A}}^{N-2}AK \end{array}\right]}}R$$

Upon applying vectorization to $R_{1}(N)$, we can obtain its vector form as follows:

(21) $$R_{1}{\left(N\right)}_{s}=(H⊗O)P_{s}+(I⊗\Gamma )R$$

Upon substituting Equation (16) into Equation (21), we readily arrive at the following expression:

(22) $$\begin{array}{c} R_{1}{\left(N\right)}_{s}=[(H⊗O){\left(I-\bar{A}⊗\bar{A}\right)}^{-1}]\\ \times (G⊗G)M\cdot q\\ +[(H⊗O){\left(I-\bar{A}⊗\bar{A}\right)}^{-1}\\ \times (AL⊗AL)+(I⊗\Gamma )]\cdot R \end{array}$$

Thus, Equation (22) establishes a linear functional relationship between the vector form of the autocorrelation sequence of the innovations and the state noise parameter vector q along with the observation noise variance R. The least squares method can be utilized to estimate the aforementioned parameters.

(23) $$\left[\begin{array}{c} q\\ R \end{array}\right]={\left(A_{\mathrm{LS}}^{T}A_{\mathrm{LS}}\right)}^{-1}A_{\mathrm{LS}}^{T}R_{1}{\left(N\right)}_{s}$$

where

(24) $$\begin{array}{l} A_{\mathrm{LS}}=[A_{Q}\;A_{R}]\\ A_{Q}=(H⊗O){\left(I-\bar{A}⊗\bar{A}\right)}^{-1}(G⊗G)M,\\ A_{R}=(H⊗O){\left(I-\bar{A}⊗\bar{A}\right)}^{-1}(AK⊗AK)+(I⊗\Gamma ). \end{array}$$

Preliminarily biased estimates of the noise parameters can affect both the convergence precision and the speed of the filter. To mitigate the impact of such biased initial values, an iterative computational strategy is commonly adopted [27]. The decision to continue iterating hinges on assessing whether the noise parameters converged. If convergence is achieved, the iteration process concludes. The specific procedure for estimating noise parameters is outlined as follows.

1. *Innovation Sequence Update*: Compute the steady-state Kalman gain and derive the sequence of innovations from the filtered measurements.
2. *Autocorrelation function*: Calculate the autocorrelation function of the innovation sequence to capture temporal correlations in the residuals.
3. *Noise parameters Estimation*: The LS method is adopted to revise the estimates of the noise parameters.
4. *Covariance Matrix Update*: Incorporate the refined noise parameter estimates to adjust and refresh the covariance matrices *Q* and *R*.
5. *Convergence Assessment of Noise Covariance Matrices*: Evaluate whether *Q* and *R* converged, indicating the stability of their values. If convergence is attained, the iterative process is concluded; otherwise, the algorithm reverts to Step 3 to iterate further.

**Figure 3:**
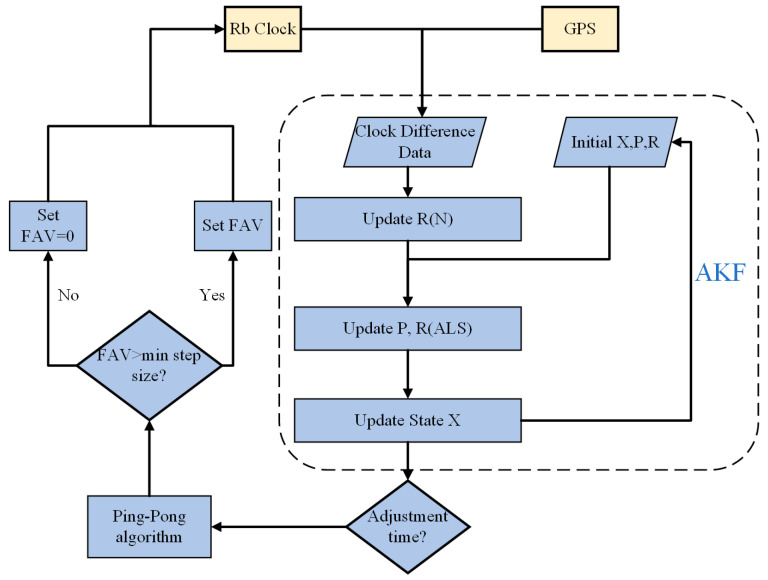
The system algorithm flowchart.

The ALS method is a correlation-based adaptive Kalman filtering approach. Its primary function is to achieve adaptive estimation of noise parameters by iteratively measuring the autocorrelation function of new information sequences, thus addressing uncertainties in system states and measurement noise. It is important to note that the adjustment interval for the rubidium clock is fixed; every hour, the ping-pong algorithm calculates the frequency adjustment value (FAV) based on the state estimate. If the FAV exceeds the predetermined minimum adjustment step size, the rubidium atomic clock is adjusted via the serial port. During each adjustment of the rubidium atomic clock, continuously acquired clock difference data are used to update the autocorrelation sequence, and the ALS method subsequently updates the new information sequence and noise parameters. Through this process, the AKF achieves adaptive estimation of noise parameters. The system algorithm flowchart is as follows (See Figure 3):

### 3.2. Initializing the State Vector

Like normal Kalman filters, adaptive Kalman filters necessitate the initialization of the state vector and the error covariance matrix. As previously mentioned, the estimation of noise parameters also requires the provision of an initial state vector. Currently, there is no universally agreed-upon scheme for selecting these initial values. It is well-known that rubidium atomic clocks exhibit excellent short-term stability. Over brief periods, the frequency deviation and clock difference of a rubidium atomic clock change minimally. By estimating the state parameters of a rubidium atomic clock based on the clock difference between the rubidium atomic clock and the reference 1PPS from the GPS, we initialize the state vector for the rubidium atomic clock as follows:

(25) $$X_{0}={\left[\begin{array}{ccc} x_{1} & y_{1} & z_{1} \end{array}\right]}^{T}$$

where

(26) $$\begin{array}{l} x_{1}=x(3)\\ y_{1}=\frac{1}{2}\left[\frac{x(3)-x(1)}{2\tau }+\frac{x(2)-x(1)}{\tau }\right]\\ z_{1}=\frac{1}{\tau }\left[\frac{x(3)-x(2)}{\tau }-\frac{x(2)-x(1)}{\tau }\right] \end{array}$$

### 3.3. Initializing the Covariance Matrix

For the initialization of the covariance matrix P, according to [28], a classical algorithm is employed, utilizing the computation formula illustrated below.

(27) $$P=\left[\begin{array}{ccc} R & \frac{3}{2\tau }R & \frac{1}{\tau }R\\ \frac{3}{2\tau }R & \frac{13}{4\tau^{2}}R+\frac{5}{2\tau }q_{1}+\frac{\tau }{3}q_{2}+\frac{\tau^{3}}{12}q_{3} & \frac{6}{\tau^{3}}R+\frac{2}{\tau^{2}}q_{1}+\frac{1}{6}q_{2}+\frac{9\tau^{2}}{40}q_{3}\\ \frac{1}{\tau }R & \frac{6}{\tau^{3}}R+\frac{2}{\tau^{2}}q_{1}+\frac{1}{6}q_{2}+\frac{9\tau^{3}}{40}q_{3} & \frac{6}{\tau^{4}}R+\frac{2}{\tau^{3}}q_{1}+\frac{2}{3\tau }q_{2}+\frac{23\tau }{40}q_{3} \end{array}\right]$$

## 4. Test Results and Analysis

### 4.1. Values of Noise Parameters

**Figure 4:**
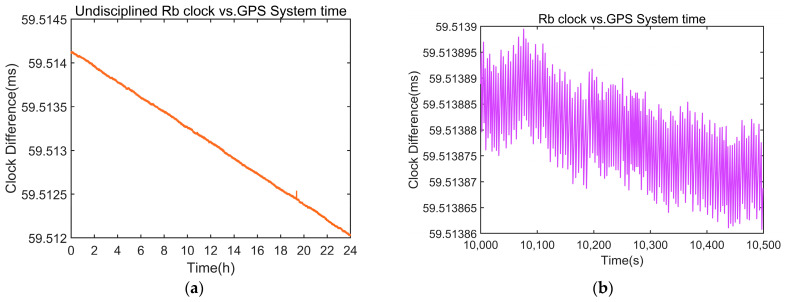
Measured clock difference data between the rubidium atomic clock and GPS before disciplining. (**a**) Clock difference data for one day. (**b**) Detailed view of the clock difference for sampling points 10,000 to 10,500.

The rubidium atomic clock necessitates a warm-up period of approximately 7 to 8 min following activation. Upon completion of this warm-up, a time interval counter is employed to measure the clock difference between the rubidium atomic clock 1PPS signal and the GPS 1PPS signal over a 24 h duration. Given the counter’s 1 Hz sampling frequency, a total of 86,400 samples were collected. The clock difference data before disciplining are shown in Figure 4.

The noise parameters of the rubidium atomic clock are estimated using the aforementioned clock difference data. The initial state noise vector is chosen as $q=\left[\begin{array}{ccc} 1 & 1 & 1 \end{array}\right]$, meaning $q_{1}=1,q_{2}=1,q_{3}=1$. The initial value for the process noise coefficient vector R is set to 5. These a priori biased initial noise parameters significantly deviate from the true noise parameters of the rubidium atomic clock. As previously mentioned, the iterative strategy effectively mitigates the impact of a priori biased initial noise parameters. Here, we specify the number of iterations to be 50.

**Figure 5:**
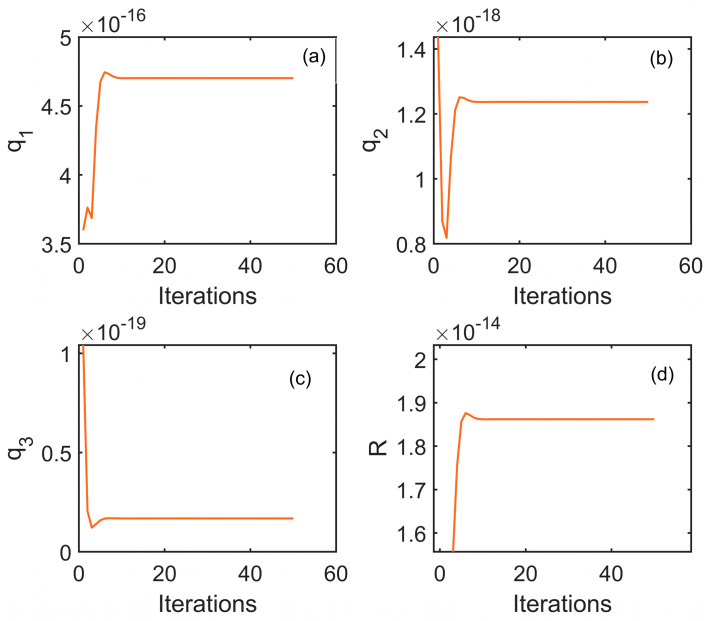
Diagram of the convergence results of the state noise parameters and process noise parameter through iteration. (**a**) $q_{1}$; (**b**) $q_{2}$; (**c**) $q_{3}$; (**d**) R.

Figure 5 and Table 1 present the noise estimation results of the rubidium atomic clock before adjustment, as estimated by the ALS algorithm. The AKF is capable of adapting its noise model to better match the current system state at each iteration step by employing the ALS algorithm, thereby achieving adaptive noise estimation and enhancing the reliability of predictive outcomes.

### 4.2. Estimation of the State Vector

To estimate the state vector of the rubidium atomic clock, we measured the clock difference data for the next hour using the counter and processed these data with the AKF. The state vector of a rubidium atomic clock typically includes three state variables: clock difference, frequency deviation, and frequency drift. The frequency drift of a rubidium atomic clock is generally very small and cannot be directly adjusted. Moreover, as indicated by the frequency control method, the frequency adjustment is based on the estimated results of the clock difference and frequency deviation of the rubidium atomic clock. The accuracy of these estimations directly affects the reliability of the frequency adjustment. As such, we focus intently on these two variables.

**Figure 6:**
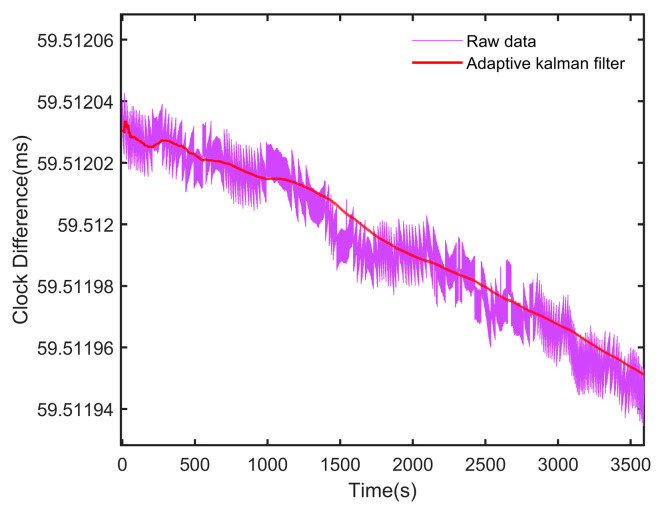
Measured clock difference and predicted values using the AKF.

Figure 6 shows the comparison between the measured clock differences and those predicted by the AKF. The clock difference predicted by the AKF is relatively minor, effectively reducing the sawtooth errors in the GPS 1PPS signal and aligning more closely with the actual clock difference of the rubidium atomic clock.

**Figure 7:**
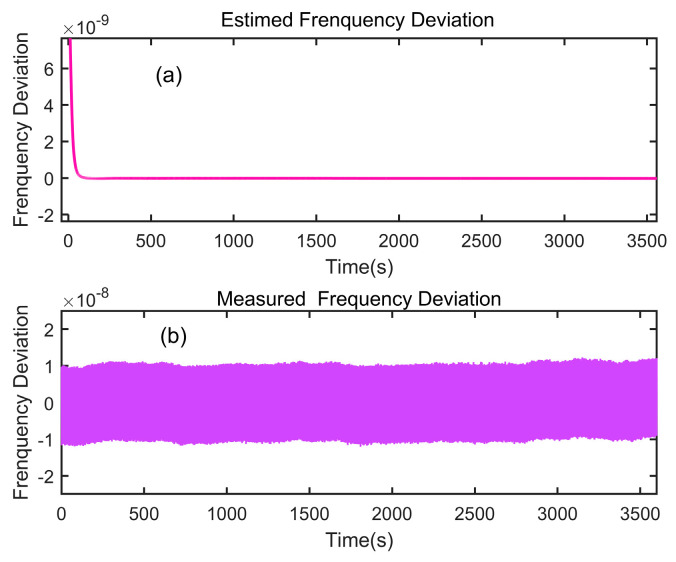
Estimated frequency deviation using the AKF and measured frequency deviation. (**a**) Estimated frequency deviation; (**b**) measured frequency deviation.

Figure 7 shows the estimation results concerning the frequency deviation of the rubidium atomic clock. It presents the frequency deviation estimation outcomes utilizing the AKF, where after a series of iterations, the estimation converges to 2.297 × 10^−11^. The LS method has a larger time lag but a high estimation accuracy. The current frequency deviation is estimated to be 2.289 × 10^−11^. Evidently, the AKF proposed in this study demonstrates high estimation accuracy.

The rubidium atomic clock interfaces with a microcontroller through a serial communication port. Utilizing a ping-pong algorithm, we translate the computed clock discrepancies and frequency deviations into precise frequency tuning directives. These directives are then dispatched as adjustment commands via the serial port, facilitating the initial calibration of the rubidium atomic clock. Thereafter, by continuously monitoring the clock’s deviation, the control system for the rubidium atomic clock is enabled to make timely frequency adjustments. This ensures that the 1PPS signal of the rubidium atomic clock is accurately synchronized with the reference 1PPS signal emitted by the GPS.

### 4.3. Disciplining Results of the Rubidium Atomic Clock

For the analysis of the disciplining effect on the rubidium atomic clock, the focus is on three pivotal metrics: firstly, examining the improvement in frequency accuracy and stability of the 10 MHz signal of the rubidium atomic clock; and secondly, evaluating the mean and variance in the clock difference between the 1PPS signal output by the rubidium atomic clock and the reference 1PPS signal.

**Figure 8:**
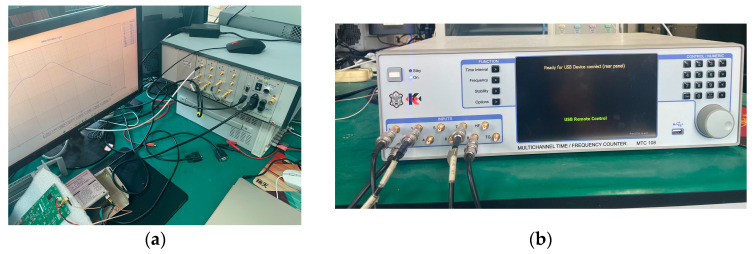
Test equipment and test site (room temperature). (**a**) Multichannel frequency comparator VCH-315; (**b**) multichannel time counter (MTC108).

The frequency accuracy and stability of the disciplined rubidium atomic clock were measured using a VCH315 instrument (Nizhny Novgorod, Russia), with the reference frequency traceable to UTC (NTSC), ensuring the precision of the evaluation benchmark. For the assessment of the performance of the disciplined rubidium atomic clock 1PPS signal, we used a high-precision multichannel time counter (MTC108) (Poznań, Poland) to measure the clock difference between the 1PPS output of the disciplined rubidium atomic clock and UTC (NTSC) 1PPS, with the counter boasting a measurement accuracy of 1.98 ps. See Figure 8.

**Figure 9:**
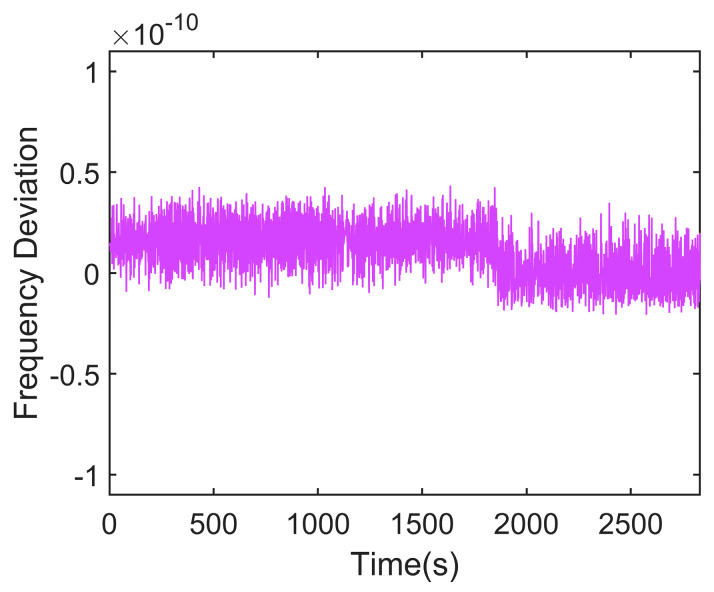
Frequency variation curve of the rubidium atomic clock before and after the initial adjustment.

We tested the frequency deviation values of the rubidium atomic clock before and after the initial adjustment, as shown in Figure 9. The test results indicated that the rubidium atomic clock can quickly adjust its frequency. After the initial adjustment, the frequency deviation of the rubidium atomic clock decreased from the order of $10^{-11}$ to the order of $10^{-12}$, and the rubidium atomic clock entered a relatively disciplined state.

**Figure 10:**
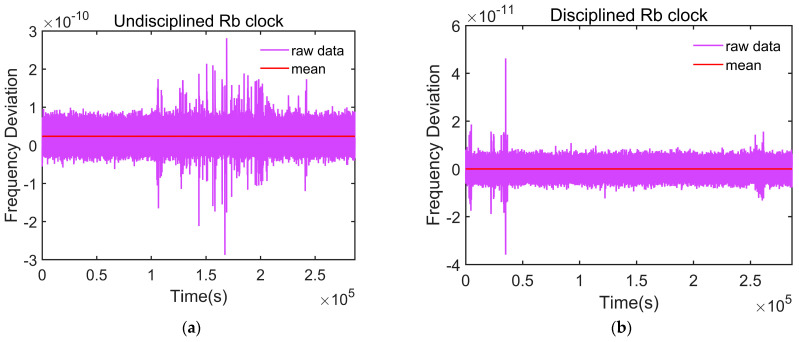
Diagram of the frequency deviation of the rubidium atomic clock before and after discipline, with the reference frequency from UTC (NTSC). (**a**) Before discipline; (**b**) after discipline.

Figure 10 shows the frequency deviation from the reference frequency before and after the rubidium atomic clock is disciplined. The mean frequency deviation of the disciplined rubidium atomic clock is 1.85 × 10^−15^, with a daily frequency drift of approximately 4.39 × 10⁻¹⁵. The frequency drift is nearly zero, making its performance comparable to that of a cesium clock. In contrast, the free-running rubidium atomic clock exhibited significant frequency drift, with a mean frequency deviation of 2.38 × 10^−11^ and a daily frequency drift of approximately $\;6.68\times 10^{-13}$. After disciplining, the frequency accuracy of the rubidium atomic clock is significantly improved.

**Figure 11:**
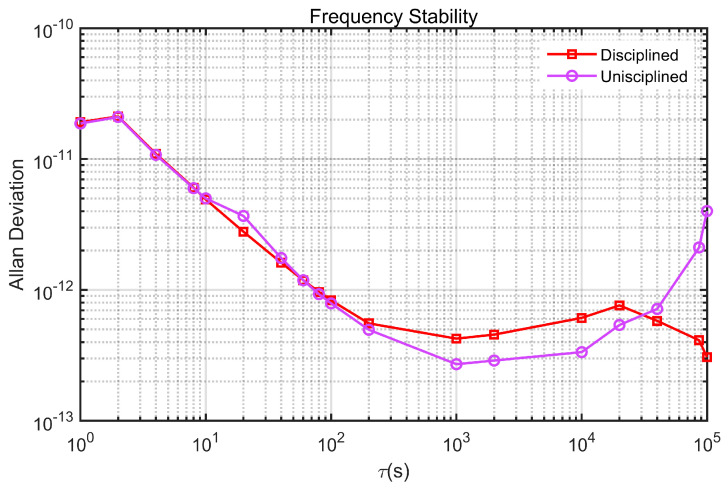
Frequency stability of the rubidium atomic clock compared to the undisciplined rubidium atomic clock.

Another important metric of the rubidium atomic clock is frequency stability, which is generally represented using the Allan deviation. We further analyzed the frequency stability of the disciplined rubidium atomic clock. To ensure the reliability of the test results, the Allan deviation measurements before and after disciplining were conducted after the clock was thoroughly warmed up, ensuring a consistent temperature. Figure 11 presents the frequency stability curves for both the disciplined rubidium atomic clock and the free-running rubidium atomic clock. It is evident from the figure that the disciplined rubidium atomic clock maintains good short-term stability. And it achieved daily stability of 4.12 × 10^−13^ and stability of 3.06 × 10^−13^ at 100,000 s. These results demonstrate that the long-term stability of the rubidium atomic clock was significantly improved through discipline.

Table 2 presents the Allan deviation of the rubidium atomic clock across various averaging timescales. A detailed comparison of the data in Figure 11 and Table 2 reveals that although the long-term stability of the rubidium atomic clock improves after disciplining, its medium- and long-term stability slightly decrease compared to its undisciplined state.

**Figure 12:**
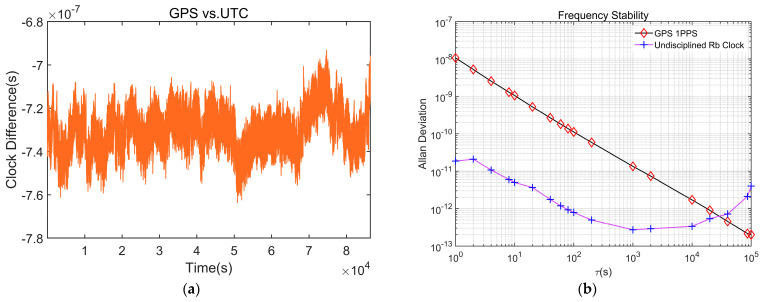
Feature analysis of GPS 1PPS. (**a**) Clock difference between the GPS 1PS and the UTC (NTSC) 1PPS signal; (**b**) frequency stability.

This phenomenon is attributed to the use of an external reference signal from GPS during the disciplining process. When the rubidium atomic clock is locked to the GPS 1PPS, the fluctuations and stability of the GPS signal will inevitably have a significant impact on the disciplining effect. To further investigate this influence, we conducted precise measurements of the clock difference between the 1PPS signal output by the GPS receiver and that of UTC (NTSC) using a counter, and analyzed its frequency stability. The graph clearly demonstrates that the 1PPS signal from GPS experiences periodic fluctuations, with a jitter magnitude of about 40 nanoseconds and a cycle period of several thousand seconds. Analysis of the frequency stability curve indicates that the GPS’s medium and short-term frequency stability is inferior to that of the rubidium atomic clock, while its long-term stability is superior. See Figure 12.

**Figure 13:**
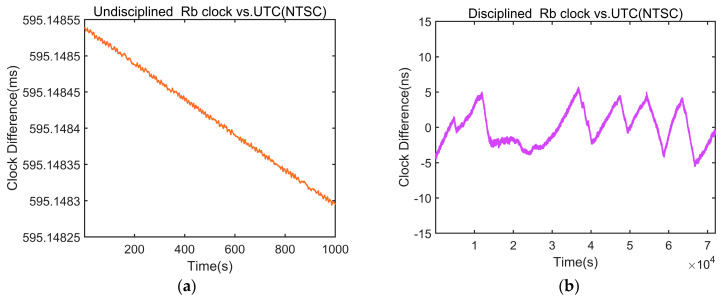
Clock difference diagram between the rubidium atomic clock and the UTC (NTSC) 1PPS signal before and after discipline. (**a**) Undisciplined rubidium atomic clock; (**b**) disciplined rubidium atomic clock.

Figure 13 shows the clock difference curves. Before disciplining, the clock difference consistently drifted in one direction. Following disciplining, the mean clock difference of the disciplined rubidium atomic clock decreases to 0.068 ns, the standard deviation decreases to 2.568 ns, and the peak-to-peak value is better than 11.358 ns. These data indicate that the performance of the 1PPS output from the disciplined rubidium atomic clock becomes significantly closer to that of the UTC (NTSC) 1PPS signal.

## 5. Conclusions

In this study, we employed an adaptive Kalman filter to process the clock offset data. During each adjustment of the rubidium atomic clock, the adaptive Kalman filter refines its innovation sequence and noise coefficients using the ALS method and newly acquired clock offset data, thereby ensuring the reliability of the state estimation. Upon reaching the adjustment epoch, the state estimation results are transformed into a specific frequency adjustment through the ping-pong algorithm. This not only ensures the continuity of the phase in the rubidium atomic clock’s output signal but also effectively corrects frequency offsets and phase errors. When compared with the traditional least squares method (LS), the state estimation results of the rubidium atomic clock further substantiated the reliability of the AKF.

The test results indicate that the standard deviation in the clock difference between the 1PPS output from the disciplined rubidium atomic clock and the 1PPS output from UTC (NTSC) is better than 2.568 ns, with a peak-to-peak value better than 11.358 ns. Additionally, the frequency accuracy of the rubidium atomic clock significantly improved. While the undisciplined rubidium atomic clock exhibits noticeable frequency drift, the disciplined rubidium atomic clock achieves an average frequency deviation of $1.85\times 10^{-15}$ and a daily frequency drift of approximately $4.39\times 10^{-15}$, approaching the performance level of cesium atomic clocks. Furthermore, the long-term stability of the rubidium atomic clock was markedly enhanced. Under conditions of maintained short-term stability, the Allan deviation of the disciplined clock is $4.12\times 10^{-13}$ @86,400 s and $3.06\times 10^{-13}$ @100,000 s. However, the mid-term stability experiences a decline, and further research is required to reduce the influence of external reference signals on the mid-term stability. In conclusion, the algorithm outlined in this paper effectively enhances the frequency accuracy and long-term stability of the rubidium atomic clock, thereby furnishing reliable time-frequency signals for applications in communication, power systems, and related fields.

## Figures and Tables

**Table 1:** Estimation results of the noise parameters.

Parameters	Notation	Value
White Phase Noise	$q_{1}$	$4.70\times 10^{-16}$
White Frequency Noise	$q_{2}$	$1.23\times 10^{-18}$
Random Walk Frequency Noise	$q_{3}$	$1.68\times 10^{-20}$
Measurement Noise	R	$1.86\times 10^{-14}$

**Table 2:** The Allan deviation of the rubidium atomic clock before and after discipline.

τ(s)	Undisciplined Rubidium Atomic Clock	Disciplined Rubidium Atomic Clock
1	$1.88\times 10^{-11}$	$1.91\times 10^{-11}$
2	$2.10\times 10^{-11}$	$2.11\times 10^{-11}$
4	$1.08\times 10^{-11}$	$1.09\times 10^{-11}$
8	$5.99\times 10^{-12}$	$6.05\times 10^{-12}$
10	$5.01\times 10^{-12}$	$4.91\times 10^{-12}$
20	$3.67\times 10^{-12}$	$2.78\times 10^{-12}$
40	$1.75\times 10^{-12}$	$1.61\times 10^{-12}$
80	$9.29\times 10^{-13}$	$9.66\times 10^{-13}$
100	$7.90\times 10^{-13}$	$8.33\times 10^{-13}$
200	$4.98\times 10^{-13}$	$5.56\times 10^{-13}$
1000	$2.71\times 10^{-13}$	$4.25\times 10^{-13}$
2000	$2.89\times 10^{-13}$	$4.55\times 10^{-13}$
10,000	$3.35\times 10^{-13}$	$6.12\times 10^{-13}$
20,000	$5.40\times 10^{-13}$	$7.62\times 10^{-13}$
86,400	$2.11\times 10^{-12}$	$4.12\times 10^{-13}$
100,000	$4.01\times 10^{-12}$	$3.06\times 10^{-13}$

## Data Availability

The supporting data for this study can be obtained upon request from the corresponding author. Due to privacy concerns involving the participants, these data are not publicly available.
